# Synthesis of Various TiO_2_ Micro-/Nano-Structures and Their Photocatalytic Performance

**DOI:** 10.3390/ma11060995

**Published:** 2018-06-12

**Authors:** Anquan Deng, Yufu Zhu, Xin Guo, Lei Zhou, Qingsong Jiang

**Affiliations:** 1 Faculty of Mechanical & Material Engineering, Huaiyin Institute of Technology, Huaian 223003, China; deng_anquan@163.com (A.D.); guoxin@hyit.edu.cn (X.G.); 2 College of Material Science and Engineering, Nanjing Tech University, Nanjing 21009, China; 3 Faculty of Mathematics and Physics, Huaiyin Institute of Technology, Huaian 223003, China; leizhou@hyit.edu.cn; 4 Jiangsu Engineering Laboratory for Lake Environment Remote Sensing Technologies, Faculty of Electronic Information Engineering, Huaiyin Institute of Technology, Huaian 223003, China; jiangqingsong05@hyit.edu.cn

**Keywords:** TiO_2_, different morphologies, photocatalytic activity, formation mechanisms

## Abstract

TiO_2_ micro-/nano-structures with different morphologies have been successfully synthesized via a hydrothermal method. The effects of the solvents on the morphology and structure of the obtained products have been studied. The objective of the present paper is to compare the photocatalytic properties of the obtained TiO_2_ products. During the synthesis process, the tetrabutyl titanate and titanium (IV) fluoride were used as the titanium source. The obtained micro-/nano-structures were characterized by field-emission scanning electron microscopy, X-ray diffraction analysis, and nitrogen adsorption-desorption isotherms. The photocatalytic activity of the samples was evaluated by the degradation of Rhodamine B solution under simulated solar irradiation. It is found that the morphologies and structures of TiO_2_ have a great influence on its photocatalytic activity. Compared with other samples, TiO_2_ flower clusters assembled with nanorods exhibited a superior photocatalytic activity in the degradation of Rhodamine B.

## 1. Introduction

In the past few decades, the synthesis of semiconductor micro-/nano-structures has attracted much attention in materials science communities due to the fact that this kind of material has many excellent properties, such as large specific surface area, high reactivity, strong adsorption and photocatalysis [1,2,3,4]. Among these materials, nano-TiO_2_ semiconductor has been considered an ideal material to solve the environmental pollution problem due to its high stability, non-toxicity, and other attributes [5,6]. A large number of studies have demonstrated that there is a close relationship between the morphological characteristics and the photocatalytic efficiencies of TiO_2_ [7,8,9]. By controlling the surface morphology and microstructure of TiO_2_, the photocatalytic performance of TiO_2_ can be enhanced. For example, I. S. Grover et al. [8] found that the photocatalytic activity of TiO_2_ nanotube crystals was superior to the commercial TiO_2_ (P25), owing to its largest surface area and hollow porous surface structure.

Recently, nano-TiO_2_ with various morphologies, including microspheres [10,11], nanosheets [12], nanorods [13], nanowires [14,15], nanotubes [15,16] and mesoporous [17], has been synthesized. Nano-TiO_2_ with these morphologies usually has a larger specific surface area and higher degree of crystallinity [10,11,12,13,14,15,16,17,18,19]. Among the above-mentioned various TiO_2_ micro-/nano-structures, mesoporous TiO_2_ has become a popular topic due to its uniform pore size, regular pore distribution and large surface area [18,20,21,22]. The previous studies have reported that the photocatalytic properties of mesoporous TiO_2_ with different morphologies are quite different, and the photocatalytic activities are significantly enhanced compared with the commercial TiO_2_ (P25) [23]. Therefore, the morphologies of TiO_2_ should have a great influence on its photocatalytic activity. However, there have been few reports on the systematic analyses and comparisons of photocatalytic properties of TiO_2_ with different morphologies and structures.

In this work, TiO_2_ with various morphologies (microspheres, flower clusters assembled with nanorods, hexahedral and nanosheet structures with different morphologies) were hydrothermally synthesized by using different titanium sources under different reaction conditions. The morphologies, sizes, and elemental analysis of the synthesized samples were examined by using field emission scanning electron microscopy (FE-SEM) and X-ray diffractometer (XRD). The photocatalytic activities of obtained products were systematically studied and compared by the degradation of Rhodamine B.

## 2. Experimental Section

### 2.1. Materials

All chemicals used in this paper are of analytical grade reagents and used without further purification [24]. Titanium tetrafluoride (TiF_4_, 99%) was purchased from J&K Scientific Ltd. (China). Hydrochloric acid (HCl, 37%), absolute ethanol, silica spheres (50 nm), and sodium hydroxide (NaOH, 96%) were purchased from Sinopharm Chemical Reagent Co., Ltd. (China). Ionic liquid (1-butyl-2,3-dimethylimidazolium tetrafluoroborate, 97%) and tetrabutyl titanate (Ti(OC_4_H_9_)_4_, TBOT, 99%) were purchased from Shanghai Aladdin Bio-Chem Technology Co., Ltd. (Shanghai, China).

### 2.2. Preparation of TiO_2_ Microspheres

In a typical synthesis, tetrabutyl titanate (TBOT) was employed as the titanium source. Primarily, 9.3 mL of absolute ethanol was mixed with 0.23 mL of HCl, followed by adding 1.3 mL of TBOT to the above mixed solution at room temperature. The whole process was carried out under the condition of magnetic stirring. After 30 min of agitation, the mixture solution was transferred to a Teflon-lined autoclave and heated at 150 °C for 4 h, then cooled down to room temperature. The final precipitate was collected by centrifugation (4000 r.p.m. for 15 min) and washed several times using absolute ethanol [25], then dried at 60 °C overnight and calcined at 400 °C for 30 min (the products were denoted as Ms). The detailed chemical reagents and reaction time used in the experiments were listed in Table 1.

### 2.3. Preparation of TiO_2_ Flower Clusters Assembled with Nanorods

During the synthesis process, TBOT was also employed as the titanium source. Hydrochloric acid and deionized water (volume ratio of 1:1) were firstly mixed by magnetic stirring, and then 0.67 mL of TBOT was added to the mixed solution. 6 mg of titanium dioxide powders was sufficiently dispersed in the above mixed solution under ultrasound irradiation. Finally, the sealed Teflon-lined autoclave containing above mixed solution was heated at 150 °C for a period of time (Table 2). The white precipitate was collected by centrifugation and washed with deionized water and absolute ethanol. Finally, the products were calcined at 400 °C for 30 min (the samples were denoted as Ns).

### 2.4. Preparation of TiO_2_ Hexahedral and Nanosheet Structures with Different Morphologies

Different morphologies of TiO_2_ hexahedral and nanosheet structures could be synthesized hydrothermally using a modified version of the procedure [26,27,28]. 40 mM or 200 mM TiF_4_ aqueous solution and 180 mM ionic liquid aqueous solution (1-butyl-2, 3-dimethylimidazolium tetrafluoroborate) were prepared in deionized water. 25 mL of TiF_4_ aqueous solution was fully mixed with a certain amount of HCl, and then, 1.5 mL of ionic liquid aqueous solution (180 mM) and 0–0.325 g silica spheres were added to the mixed solution with magnetic stirring. The above solutions were transferred to a Teflon-lined autoclave and heated at 170 °C for a certain amount of time. Details describing the synthesis of the products can be found in Table 3. The obtained white precipitate was washed several times with deionized water. The silica template in the precipitate was selectively etched in aqueous 2M NaOH at 80 °C for 1 h [27]. The prepared samples were calcined at 400 °C for 30 min. The yielded products were respectively denoted as S1, S2, and S3, as demonstrated in Table 3.

### 2.5. Characterization

The morphologies of the obtained samples were examined by using field emission scanning electron microscopy (FESEM; Quanta 250 FEG, FEI, America) with an accelerating voltage of 15 kV. The samples were coated with a layer of gold by using vacuum sputtering coating machine (Q150R ES, UK) before morphological characterization. Phase analysis of the samples was recorded on a Bruker AXS Advanced X-Ray diffractometer system (XRD; D8 Advance, Bruker, Germany) in the 2θ scan range of 20–80°. N_2_ adsorption–desorption isotherms were recorded at 77 K using a Micromeritics Tristar 3020 analyzer (Micromeritics Instrument Corp., Norcross, GA, USA).

### 2.6. Photocatalysis Measurements

Under simulated solar irradiation, the photocatalytic measurement of the obtained samples was carried out in a 100 mL glass photoreactor. The distance between fixed intensity light source and photoreactor is 13 cm. The specific experimental procedures are as follows: 0.03 g sintered samples were added into the glass photoreactor containing 25 mL of Rhodamine B solution (10 mg L^−1^) and 0.5 mL of 30% of H_2_O_2_. With constant stirring, the above photoreactor was placed in the dark environment for 1 h, and then the reaction liquid was irradiated with a 300 W Xenon lamp (PLS-SXE300C, λ = 300–2500 nm, Beijing Perfectlight Technology Co., Ltd., Beijing, China). At set intervals, a small amount of reaction liquid was withdrawn and centrifuged. The obtained supernatant was preserved for the subsequent analysis. Then the absorbance of supernatant was detected at wavelengths of 450~600 nm by an UV-visible spectrophotometer (Shimadzu UV-3600, Shimadzu corporation, Kyoto, Japan). Simultaneously, some control experiments were also carried out under the condition that only light, only H_2_O_2_, or only catalyst and light was applied during the photocatalytic process.

## 3. Results and Discussion

### 3.1. Characterization of TiO_2_ Microspheres

**Figure 1:**
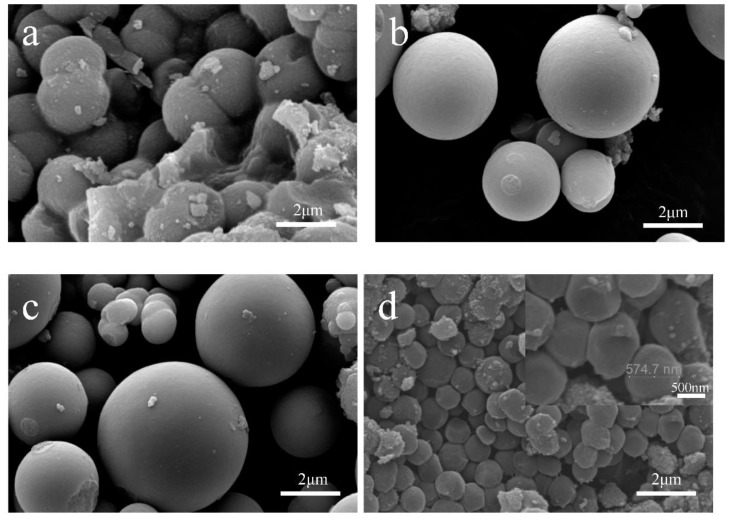
Field Emission Scanning Electron Microscopy (FESEM) images of Ms prepared with different amounts of HCl: (**a**) 0 mL; (**b**) 0.03 mL; (**c**) 0.43 mL and (**d**) 1.50 mL.

Compared with other structures, the microspheres have been widely concerned for its unique morphology [29]. Moreover, the pH value and solvent dosage have a great influence on the morphology of products synthesized by the hydrothermal method. In order to investigate the effect of the pH value on the morphology of TiO_2_ microspheres, TiO_2_ with different morphologies were prepared by changing the amounts of HCl in the reaction solution. The SEM images of the obtained products were shown in Figure 1. From Figure 1a, it can be clearly seen that the size of the as-obtained TiO_2_ microspheres are uniform and the microspheres are aggregated without the addition of HCl in the reaction solution. With increasing the amount of HCl to 0.03 mL and 0.43 mL, the microspheres with smooth surface can be obtained, and the sizes of microspheres are increased (Figure 1b,c). When 1.5 mL of HCl was added in the reaction solution, small-sized TiO_2_ spheres with an average diameter of approximately 500 nm were obtained, as demonstrated in Figure 1d. Based on the above results, it indicates that the amount of HCl added in the reaction solution can be used to control the morphologies and sizes of the products. With increasing HCl dosage, the hydrolysis rate of TBOT was inhibited, so the synthesized samples have good dispersibility. However, when HCl dosage in the reaction solution is too high, the growth of crystal grains will be hindered, resulting in the agglomeration of particles and reduction of size.

**Figure 2:**
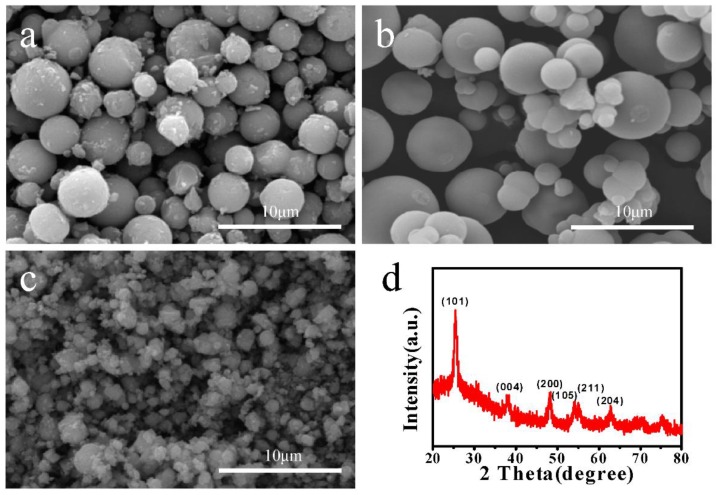
FESEM images of Ms prepared with different amounts of absolute ethanol: (**a**) 4.65 mL; (**b**) 9.30 mL and (**c**) 18.6 mL; (**d**) XRD pattern of Ms.

Figure 2 shows SEM images and XRD pattern of the TiO_2_ microspheres prepared with different amounts of absolute ethanol. When the amount of absolute ethanol was 4.65 mL, TiO_2_ microspheres with rough surface were obtained and the sizes of the microsphere were non-uniform (Figure 2a). As the amounts of absolute ethanol increased, the surface of microspheres became smooth (Figure 2b). When 18.6 mL of absolute ethanol was added in the reaction solution, the size of microspheres had shrunk dramatically, and couldn’t form a complete spherical structure (Figure 2c). These results show that the amount of ethanol has a great influence on the morphology of TiO_2_ microspheres, which can be explained by the following: with increasing the amount of ethanol, the nanoparticles generated by TBOT hydrolysis are distributed uniformly in solution, forming stable and dispersive microspheres. However, an excess of ethanol added in the solution results in a relatively low concentration of Ti source, which does not allow assembling to form spherical structures, eventually resulting in the irregular aggregates shown in Figure 2c.

To further determine the crystal structure of the obtained microspheres, the phase analysis of the samples was carried out. Figure 2d shows the typical XRD pattern of the obtained microspheres. From this figure, it can be clearly seen that the diffraction peaks are consistent with the anatase TiO_2_ (JCPDS21-1272) [30], having diffraction peaks of (101), (004), (200), (105), (211) and (204) planes at 2θ = 25.4°, 38°, 48°, 54°, 56° and 63°, respectively. Therefore, the as-obtained samples can be determined as anatase TiO_2_.

### 3.2. Characterization of TiO_2_ Flower Clusters Assembled with Nanorods

**Figure 3:**
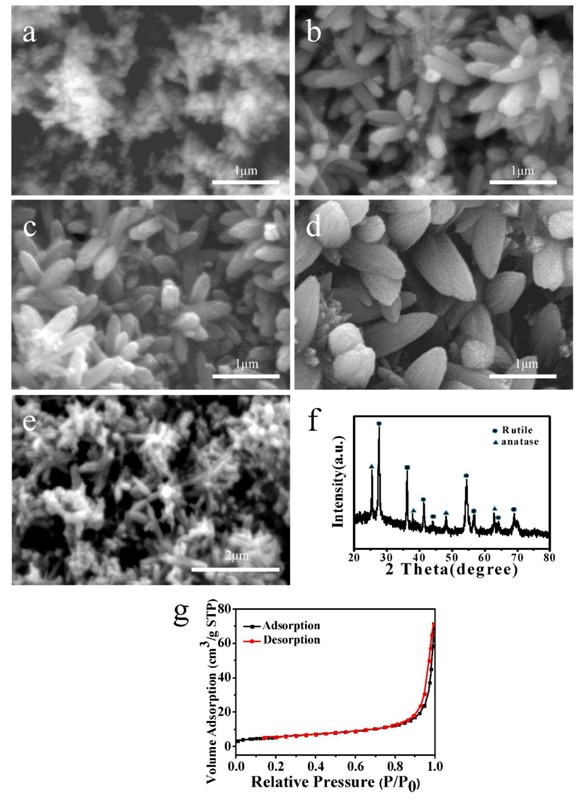
FESEM images of Ns prepared with different reaction times: (**a**) 12 h; (**b**) 18 h and (**c**) 24 h. (**d**) FESEM image of Ns prepared without the addition of titanium dioxide powders for 18 h. (**e**) FESEM image, (**f**) XRD pattern and (**g**) N_2_ adsorption-desorption isotherm of Ns calcined at 400 °C.

The related research shows that nanorods generally exhibit larger specific surface area than microspheres. Therefore, the synthesis of TiO_2_ flower clusters assembled with nanorods was achieved by a hydrothermal method. The formation process of the nanostructures was studied by controlling the hydrothermal reaction time and the addition of titanium dioxide powders in the reaction solution; the results are displayed in Figure 3a–d. As shown in Figure 3a, when the hydrothermal reaction was carried out for 12 h, TiO_2_ nanorods were not fully formed. With prolonging the reaction time up to 18 h, flower-like TiO_2_ nanostructures were formed. The average diameter and length of nanorods were approximately 300 nm and 500 nm, respectively (Figure 3b). When the reaction time was up to 24 h, the TiO_2_ nanorods were further grown and the flower-like structure was obtained (Figure 3c). The above results indicate that the reaction time determines the morphologies of the products. Figure 3d represents FESEM image of TiO_2_ flower clusters assembled with nanorods prepared without the addition of titanium dioxide powders for 18 h. Compared with the SEM image shown in Figure 3b, it can be seen that the prepared nanorods became larger in size. The reason for this phenomenon is that the added titanium dioxide powders in the reaction solution acted as nucleus for crystal growth and the Ti source depleted faster than that without titanium dioxide powders, which inhibits further growth of nanoparticles, resulting in a reduction in the size of the final nanorods.

Figure 3e is FESEM image of TiO_2_ flower clusters assembled with nanorods calcined at 400 °C. From this figure, it can be observed that the heat treatment didn’t destroy the morphology of the samples. The typical XRD pattern of the as-synthesized samples is illustrated in Figure 3f. The diffraction peaks are basically indexed to rutile TiO_2_ (JCPDS21-1276) [31]. Furthermore, the additional peaks appear at 25.4°, 38°, 48°, and 62.8°, which can be indexed to anatase TiO_2_ (JCPDS21-1272). The results of XRD indicate that TiO_2_ flower clusters assembled with nanorods were mixed crystal of rutile and anatase. From the analysis of BET surface areas of the samples, the N_2_ adsorption-desorption isotherm is shown in Figure 3g. The estimated BET surface area of the samples is 20.1 m^2^/g. The larger surface area and special morphology of flower-like structure together should contribute to the improved photocatalytic activity.

### 3.3. Characterization of TiO_2_ Hexahedral and Nanosheet Structures with Different Morphologies

**Figure 4:**
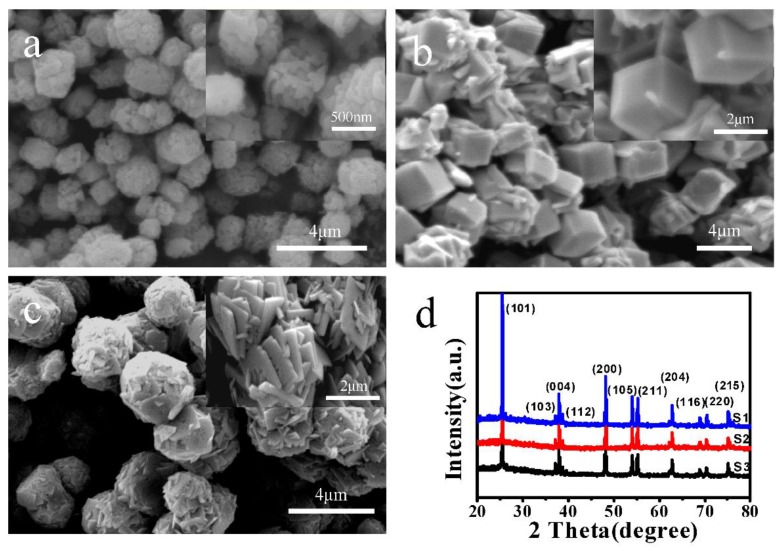
FESEM images of TiO_2_ hexahedral and nanosheet structures with different morphologies: (**a**) S1; (**b**) S2 and (**c**) S3; (**d**) XRD patterns of S1, S2, and S3.

The porosity and structural properties on the surface of the samples have a certain influence on its photocatalytic performance. Hence, in order to systematically study the relationship between morphology, structure, and photocatalytic performance of TiO_2_, the hydrothermal synthesis of hexahedral and nanosheet structures with different morphologies were also carried out. SEM images and XRD patterns of the samples are shown in Figure 4. S1 was hydrothermally synthesized by using silica spheres as template, which showed hexahedral structures with an average size of about 500 nm. The surface was very rough and presented numerous striped porous structures (Figure 4a). Its formation mechanism can be interpreted as follows: during the hydrothermal reaction process, the hydrolysis of TiF_4_ was carried out. The generated TiO_2_ nanoparticles were deposited randomly on the surface of the SiO_2_ templates. After the templates were etched by sodium hydroxide aqueous solution, the porous structures as demonstrated in Figure 4a were formed. S2 was hexahedral structures with a side length of about 2 µm, which was prepared without the addition of SiO_2_ templates (Figure 4b). Compared with S1 (Figure 4a), microstructures with relatively smoother surfaces were obtained. Figure 4c presents the low- and high-magnification SEM images of the as-prepared S3. From this figure, it can be clearly observed that TiO_2_ microspheres were composed of a number of self-assembled crystal nanosheets with a thickness of approximately 100 nm. Compared with the preparation condition of S2, the new crystal began to nucleate and grow on the nanosheets due to the increased concentration of TiF_4_. As the hydrothermal reaction proceeded, more nanosheets were produced, and finally, the self-assembled TiO_2_ microspheres with nanosheets were obtained. Figure 4d shows the XRD patterns of S1, S2, and S3. Obviously, there were similar diffraction patterns and sharp diffraction peaks. The diffraction peaks were highly consistent with that of anatase TiO_2_ (JCPDS21-1272).

### 3.4. Photocatalysis Measurement

**Figure 5:**
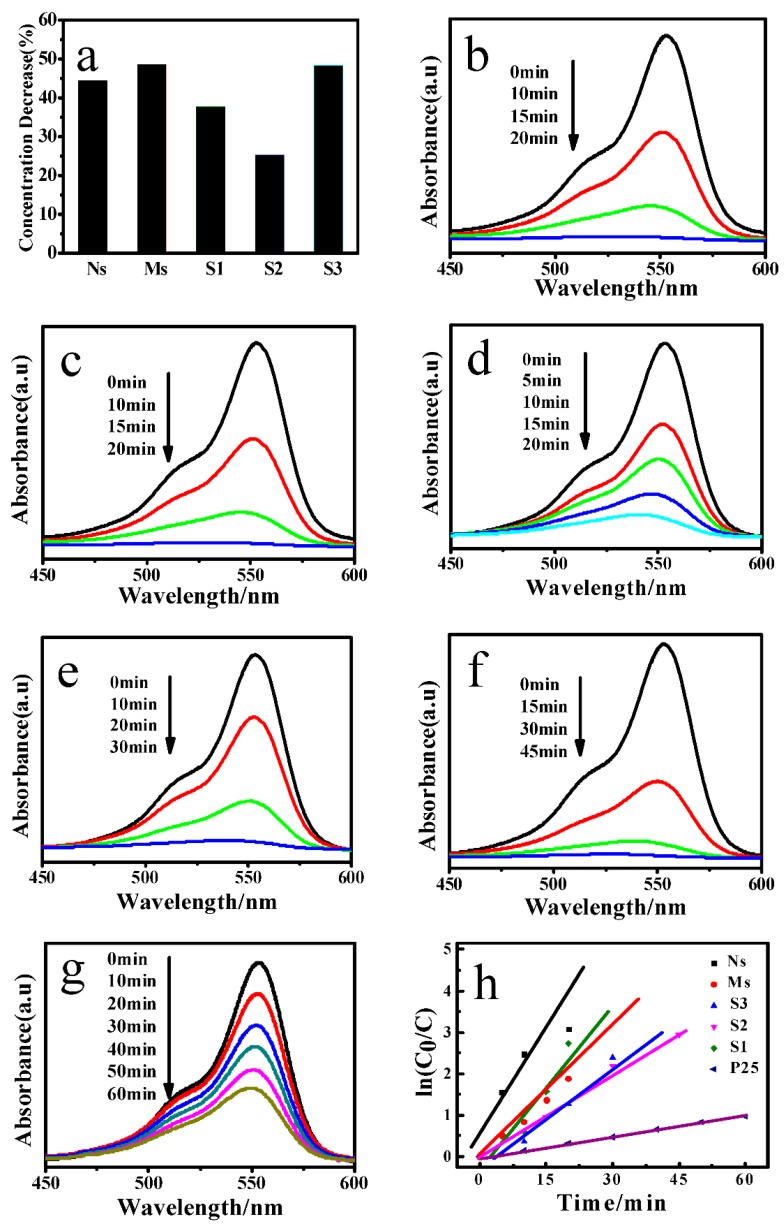
(**a**) The concentration decreases of Rhodamine B (RhB) aqueous solution in dark for 1 h before illumination; UV−vis spectra of (**b**) Ns; (**c**) S1; (**d**) Ms; (**e**) S3; (**f**) S2; (**g**) P25; and (**h**) kinetics of RhB degradation over samples.

Simulated-sunlight-induced photocatalytic degradation of Rhodamine B solution was performed to evaluate the photocatalytic activity of Ns, Ms, S1, S2, and S3. To explore adsorption properties of the samples for Rhodamine B, the obtained Ns, Ms, S1, S2 and S3 were added in the Rhodamine B solution and placed in dark for 1 h before illumination. As shown in Figure 5a, the adsorption properties of Ns, Ms, S1, S2 and S3 were different. The absorption rates for Ns, Ms, S1, S2 and S3 were 44.36%, 48.57%, 37.78%, 25.25%, and 48.27%, respectively. Among them, Ms exhibited the best adsorption properties (Ms > S3 > Ns > S1 > S2). This indicates that the morphologies and structures of the TiO_2_ affect the adsorption properties of the obtained products.

Figure 5b–g displays UV-vis spectra of Rhodamine B solution degraded by the samples. From these figures, the decrease in absorbance of Rhodamine B solution can be clearly observed along with increasing the irradiation time. In addition, compared with P25, the whole processes of photocatalytic degradation of the synthesized samples were completed in a relatively short period of time.

In order to compare the photocatalytic performances of the obtained samples more intuitively, based on UV-vis spectra displayed in Figure 5b–g, the kinetics of Rhodamine B photo-degradation over Ns, Ms, S1, S2, S3, and P25 are given in Figure 5h. The *k* and C_0_/C represent the degradation reaction rate in min^−1^ and the normalized Rhodamine B concentration, respectively [32]. Through the analysis of the kinetics, one can draw a conclusion that the order of the degradation reaction rate is listed below: Ns (*k* = 0.183) > S1 (*k* = 0.145) > Ms (*k* = 0.106) > S3 (*k* = 0.082) > S2 (*k* = 0.065) > P25 (*k* = 0.016). The results explicitly indicate that Ns have best degradation performance. Therefore, the Ns are the more suitable catalyst for the degradation of Rhodamine B.

**Figure 6:**
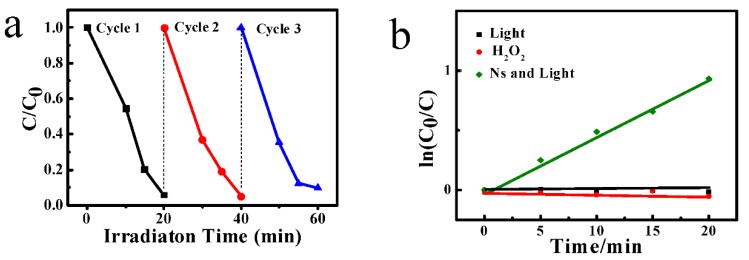
(**a**) Cyclic degradation efficiency of Ns under simulated solar irradiation; (**b**) The effect of light and H_2_O_2_ on the degradation efficiency of the catalyst.

The catalytic longevity of the catalysts was also studied, and the results are shown in Figure 6a. After three cycles of degradation of Rhodamine B solution, the degradation rate of Ns is still very high. It thus demonstrates that the obtained catalysts possess excellent catalytic longevity. In order to investigate the influence of light and H_2_O_2_ on the degradation performance of the catalysts, a series of controlled catalytic experiments were performed. As shown in Figure 6b, it can be seen that no degradation of Rhodamine B can be observed under the condition that only light or H_2_O_2_ was applied during the photocatalytic process. When light and Ns was simultaneously applied, the degradation of Rhodamine B took place. The degradation rate is lower than that of the solution containing Ns and H_2_O_2_ (Figure 5h). From the above experimental results, it can be concluded that synergistic effects of Ns, light and H_2_O_2_ contributed to the enhanced degradation efficiency.

## 4. Conclusions

TiO_2_ with different morphologies were successfully synthesized by a facile hydrothermal method. The XRD results indicate that TiO_2_ flower clusters assembled with nanorods are of a mixed crystal phase consisting of rutile and anatase. The other synthesized TiO_2_ nanostructures are anatase. Based on the photocatalytic tests, it can be seen that all as-prepared samples have good photocatalytic performance. Among these samples, TiO_2_ flower clusters assembled with nanorods exhibit superior photocatalytic activity. The order of photocatalytic efficiencies of samples is as follows, Ns > S1 > Ms > S3 > S2, indicating that the morphologies and structures of TiO_2_ have a great influence on its photocatalytic activity.

## Figures and Tables

**Table 1:** TiO_2_ microspheres (Ms) samples synthesized with chemical reagents and reaction time in the experiments.

TBOT Addition (mL)	HCl Addition (mL)	Absolute Ethanol (mL)	Reaction Time (h)
1.3	0.00	9.30	4
1.3	0.03	9.30	4
1.3	0.43	9.30	4
1.3	1.50	9.30	4
1.3	0.23	4.65	4
1.3	0.23	9.30	4
1.3	0.23	18.6	4

**Table 2:** Ns samples synthesized with chemical reagents and reaction time in the experiments.

C_6_H_36_O_4_Ti Addition (mL)	HCl Addition (mL)	H_2_O (mL)	TiO_2_ Addition (g)	Reaction Time (h)
0.67	16	16	0.006	12
0.67	16	16	0.006	18
0.67	16	16	0.006	24
0.67	16	16	0.0	18

**Table 3:** S1, S2, and S3 samples synthesized with chemical reagents and reaction time in the experiments.

Sample	TiF_4_ Addition (mmol/L)	HCl Addition (µL)	Ionic Liquid Aqueous Solution (mL)	SiO_2_ Addition (g)	Reaction Time (h)
S1	40	1000	1.5	0.325	20
S2	40	1000	1.5	0.0	20
S3	200	20	1.5	0.0	16
